# Transduction of Brain Dopamine Neurons by Adenoviral Vectors Is Modulated by CAR Expression: Rationale for Tropism Modified Vectors in PD Gene Therapy

**DOI:** 10.1371/journal.pone.0012672

**Published:** 2010-09-17

**Authors:** Travis B. Lewis, Joel N. Glasgow, Anya M. Glandon, David T. Curiel, David G. Standaert

**Affiliations:** 1 Department of Cell Biology, The University of Alabama at Birmingham, Birmingham, Alabama, United States of America; 2 Division of Human Gene Therapy, Departments of Medicine, Obstetrics and Gynecology, Pathology, and Surgery, The University of Alabama at Birmingham, Birmingham, Alabama, United States of America; 3 Center for Neurodegeneration and Experimental Therapeutics, The University of Alabama at Birmingham, Birmingham, Alabama, United States of America; 4 Gene Therapy Center, The University of Alabama at Birmingham, Birmingham, Alabama, United States of America; 5 Division of Cardiovascular Disease, Department of Medicine, The University of Alabama at Birmingham, Birmingham, Alabama, United States of America; Brigham and Women's Hospital, Harvard Medical School, United States of America

## Abstract

**Background:**

Gene-based therapy is a new paradigm for the treatment of Parkinson disease (PD) and offers considerable promise for precise targeting and flexibility to impact multiple pathobiological processes for which small molecule agents are not available. Some success has been achieved utilizing adeno-associated virus for this approach, but it is likely that the characteristics of this vector system will ultimately create barriers to progress in clinical therapy. Adenovirus (Ad) vector overcomes limitations in payload size and targeting. The cellular tropism of Ad serotype 5 (Ad5)–based vectors is regulated by the Ad attachment protein binding to its primary cellular receptor, the coxsackie and adenovirus receptor (CAR). Many clinically relevant tissues are refractory to Ad5 infection due to negligible CAR levels but can be targeted by tropism-modified, CAR-independent forms of Ad. Our objective was to evaluate the role of CAR protein in transduction of dopamine (DA) neurons *in vivo*.

**Methodology/Principal Findings:**

Ad5 was delivered to the substantia nigra (SN) in wild type (wt) and CAR transgenic animals. Cellular tropism was assessed by immunohistochemistry (IHC) in the SN and striatal terminals. CAR expression was assessed by western blot and IHC. We found in wt animals, Ad5 results in robust transgene expression in astrocytes and other non-neuronal cells but poor infection of DA neurons. In contrast, in transgenic animals, Ad5 infects SNc neurons resulting in expression of transduced protein in their striatal terminals. Western blot showed low CAR expression in the ventral midbrain of wt animals compared to transgenic animals. Interestingly, hCAR protein localizes with markers of post-synaptic structures, suggesting synapses are the point of entry into dopaminergic neurons in transgenic animals.

**Conclusions/Significance:**

These findings demonstrate that CAR deficiency limits infection of wild type DA neurons by Ad5 and provide a rationale for the development of tropism-modified, CAR-independent Ad-vectors for use in gene therapy of human PD.

## Introduction

Parkinson disease (PD) is a progressive neurodegenerative disease which causes a movement disorder characterized by bradykinesia, resting tremor, rigidity, and postural instability. These symptoms result primarily from progressive degeneration of dopamine (DA) producing neurons in the substantia nigra pars compacta (SNc). Despite progress in treating the symptoms of PD, no therapy to date has been shown to alter the course of the neurodegenerative process.

Gene-based therapy is a new paradigm for the treatment of PD and offers considerable flexibility to impact multiple pathobiological processes for which small molecule agents are not available. Gene-based approaches for PD have been evaluated in Phase I and II clinical trials [1], [2]. These studies have included both enzyme-based therapies directed towards alleviation of symptoms [3], [4], [5], [6], [7], [8], [9], and growth factor-based approaches with potential for neuroprotective and neurorestorative effects [10], [11], [12], [13]. The majority of clinical gene delivery approaches in PD have employed viral vectors based on adeno-associated virus (AAV). While some success has been achieved with the AAV approach, it is likely that the characteristics of this vector system may ultimately limit its clinical utility. Specifically, AAV vectors offer neither sufficient flexibility in targeting to enable cell specific delivery, nor sufficient genomic capacity for larger therapeutic cargoes that may prove most promising.

Use of adenovirus (Ad)-based vectors for gene delivery to the central nervous system (CNS) may provide a number of advantages over AAV-based strategies. Ad vectors can deliver up to 36kb of genetic material, providing space for both therapeutic transgenes and tissue-specific promoter elements for cell-specific transcriptional targeting. Further, the ability to modify Ad vector tropism to target specific tissues (termed transductional targeting) is well established. The combination of transcriptional and transductional targeting (double targeting) can provide a high degree of selectivity and limit off-target effects, leading to enhanced safety and efficacy [14], [15].

Ad serotype 5 (Ad5)-based adenoviral vectors have been employed for other applications, but are not well suited for PD gene therapy. When injected into the CNS, previous studies have established that Ad5-based vectors preferentially infect astrocytes to a greater extent than neurons [16], [17], [18]. In this regard, the tropism of Ad5-based vectors is determined by direct binding of the Ad5 fiber protein to its primary cellular receptor, the coxsackie and adenovirus receptor (CAR) [19], [20]. The dependence of Ad5 transduction on CAR expression results in a scenario wherein high-CAR non-target cells can be infected, whereas target tissues, if low in CAR, remain poorly infected [14], [15]. Indeed, many clinically relevant tissues are often refractory to Ad5 infection due to negligible CAR levels, but can successfully be targeted by tropism-modified Ad vectors that utilize alternate receptors [14], [15], [21], [22], [23], [24], [25].

The expression of CAR in the nervous system and the role of this protein in targeting Ad5 vectors in the brain have not been closely examined [26], [27]. In this study, we evaluated the ability of Ad5 vectors to infect dopamine neurons *in vivo* both in wild type animals and those genetically engineered to express heterologous human CAR (hCAR) [27]. We have found that Ad5 delivery to the wild type C57BL/6 mouse SN results in robust transgene expression in astrocytes and other non-neuronal cells, but poor transduction of dopaminergic neurons. In contrast, Ad5 delivery to transgenic animals expressing hCAR results in increased transduction of neurons of the SNc. Direct assessment of CAR protein in the ventral midbrain revealed that there is a relative paucity of CAR expression in this region in wild type animals, while nigral CAR expression is markedly increased in hCAR transgenic animals. These findings demonstrate the importance of tropism for the efficient transduction of SN neurons by Ad vector, and provide a rationale for the development of tropism-modified, CAR-independent Ad vectors for use in gene therapy of human PD.

## Results

### Ad5 transduction of dopamine neurons in the SN in wild type animals *in vivo*

**Figure 1:**
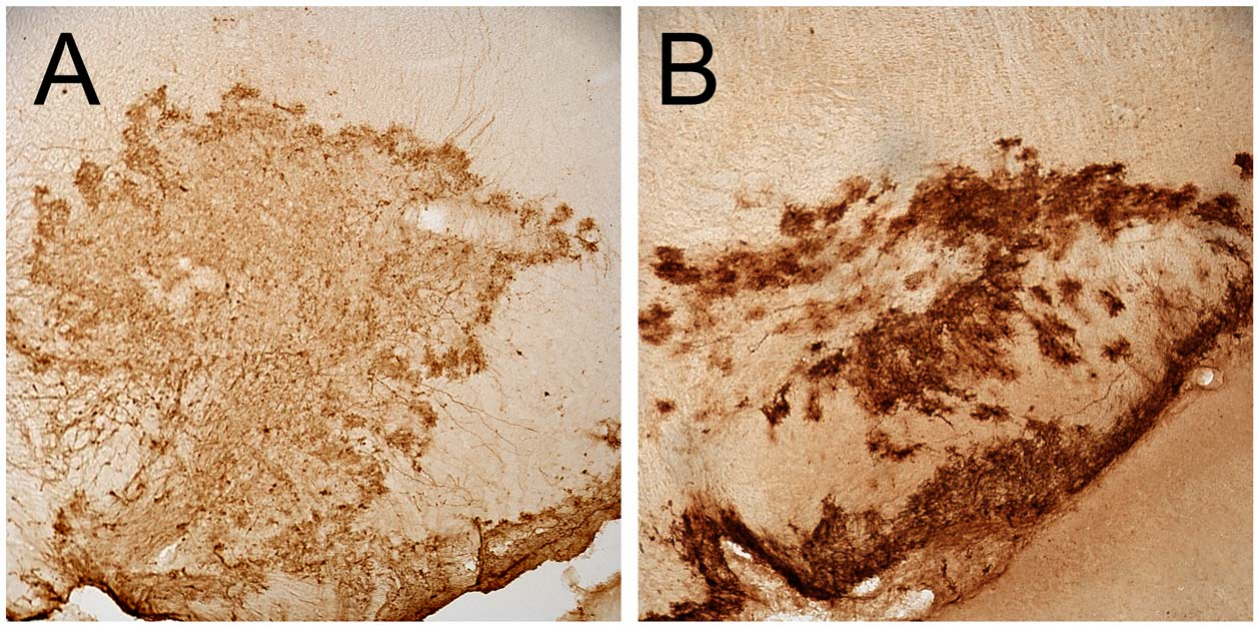
Ad5 drives high levels of transgene production. Sections through the core of the injection volume in the SN stained for the GFP transgene, demonstrate the robust levels of expression in this system. This activity made unbiased stereologic counting unfeasible. Panel **A** is SN of a wt animal. Panel **B** is SN of an hCAR transgenic animal.

**Figure 2:**
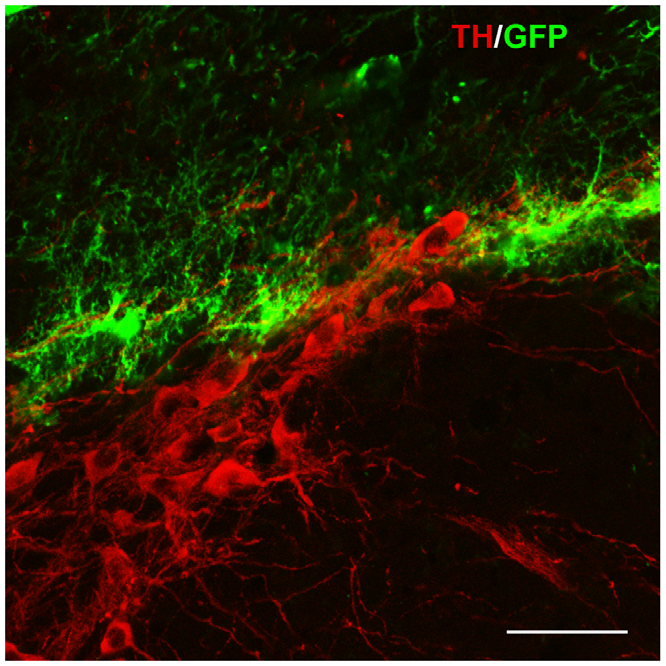
Assessment of Ad5 transduction in the SN *in vivo*. Confocal immunohistochemical assessment of 1e8 vp of Ad5-GFP delivered to the mouse SN (red) shows broad transgene expression in cells of glial morphology (green). The absence of double-labeled cells indicates Ad5 poorly infects neurons of the SNc (section through periphery of the injection site). Stereotactic coordinates: AP = 3.1, ML = −1.2, DV = −4.6. Red  =  TH–positive neurons, Green  =  GFP. Bar  = 50 µm.

We assessed the ability of a replication deficient Ad5 vector encoding green fluorescent protein (GFP) to infect neurons following stereotaxic injection into the SNc. Immunohistochemical staining for GFP and tyrosine hydroxylase (TH), a marker of dopamine neurons, was performed either 7 or 14 days after vector delivery. In each of eight animals examined, we detected strong GFP expression in the region of the SNc. In the core of the injection site, there was extensive labeling of neural and glial structures with confluent labeling that made distinguishing individual cellular profiles difficult (**Figure 1A**). Towards the periphery of the injection site, the cellular localization of the staining could be resolved, and the majority of the GFP-positive cells were small with astrocytic morphology (**Figure 2**). Few GFP-positive neurons were detected and only rarely were GFP and TH double positive neurons observed in wild type animals.

### hCAR transgenic animals exhibit enhanced transduction of dopamine neurons by Ad5 vectors

**Figure 3:**
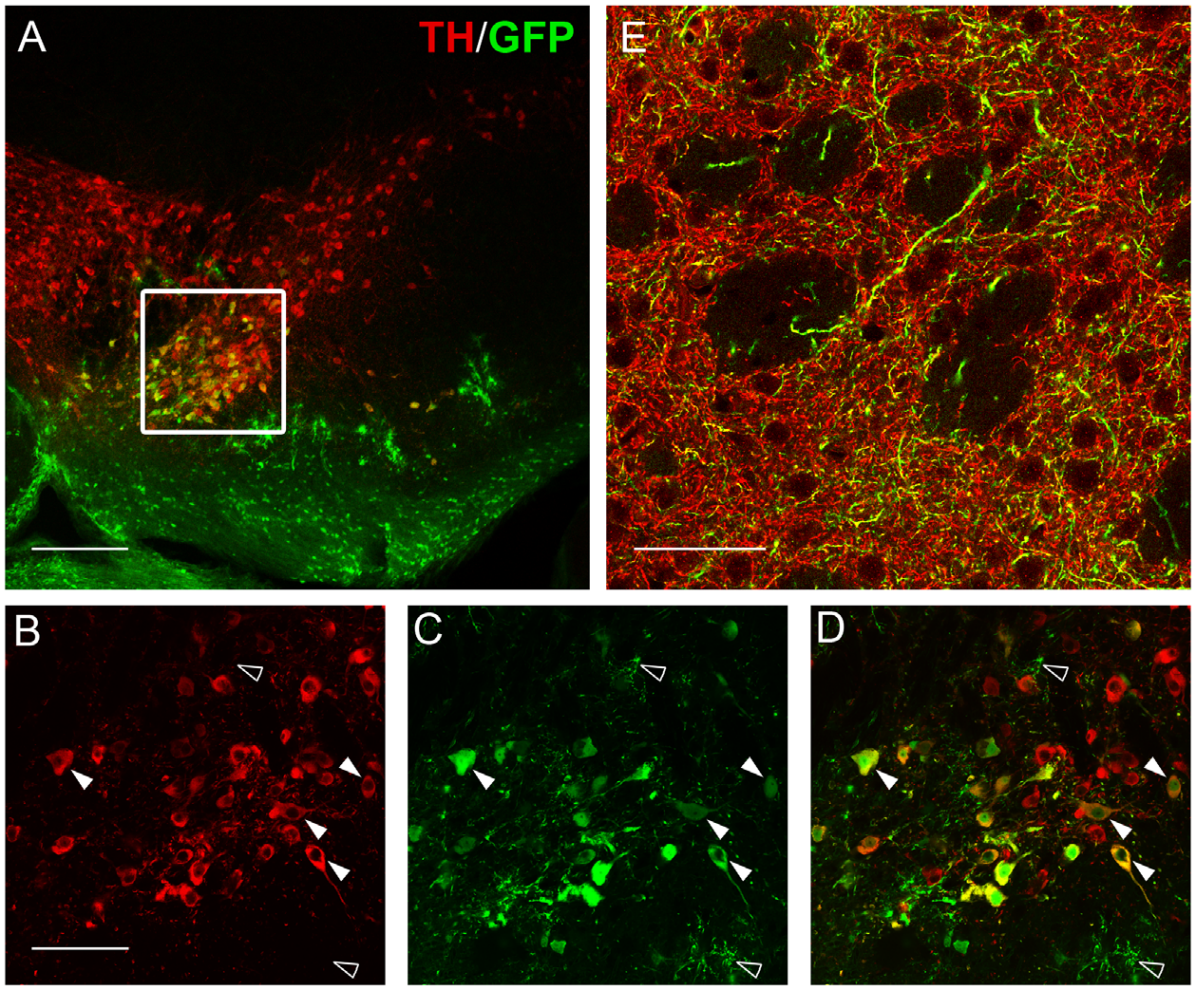
hCAR transgenic animals exhibit transduction of dopamine neurons by Ad5 vectors. Delivery of Ad5 to the SN in the hCAR mouse shows a broadened transduction profile including TH+ neurons by confocal microscopy. Transgene expression extends into the STR terminal fields. (**A**) Low power image of the hCAR SN shows a broadened transduction profile of Ad that includes TH-positive neurons, in addition to astrocytes. Magnification of the region outlined in **A** shows many TH-positive cells (red, **B**) staining positive for GFP (green, **C**) overlaid in **D** (see closed arrowheads). GFP-positive cells with astrocyte morphology can also be seen in this region (open arrowheads). (**E**) Visualization in the STR shows many fibers labeling with GFP, indicating their transduction by Ad5 delivered in the SNc. Red  =  TH-positive neurons, Green  =  GFP, Yellow  =  double positive. Scale bar **A**: 500 µm, **B–D**: 100 µm, **E**: 50 µm.

To better understand the role of CAR in Ad5 transduction of brain cells, we delivered Ad5 encoding GFP to the SNc of eight transgenic animals expressing hCAR and eight wild type (wt) controls. As in the wt animals, in hCAR animals we observed extensive transduction in the region of the SNc; at the core of the injection site the labeling was essentially confluent, with staining of many cells with astrocytic morphology (**Figure 1B**). At the margins of the injection site, however, we found that in hCAR animals there was also substantial transduction of TH-positive neurons within the SNc (**Figure 3A–D**). Furthermore, we observed GFP-positive fibers in the ascending medial forebrain bundle and striatum, a feature rarely observed in the wild type animals (**Figure 3E**).

**Figure 4:**
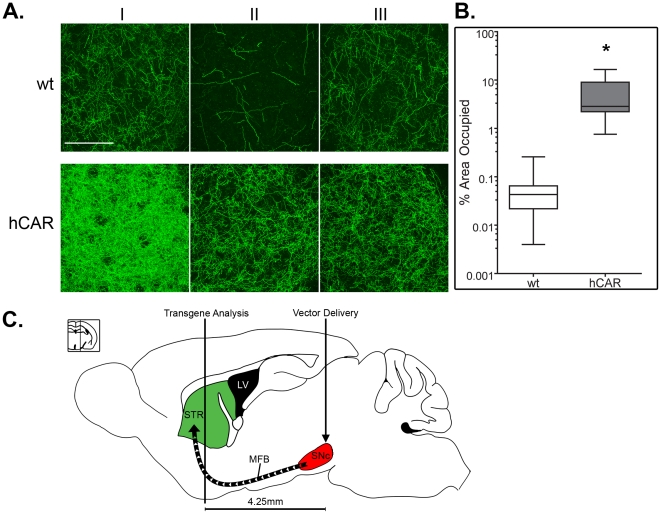
Quantitation of transgene in the striatal terminal fields. Densitometric analysis of transgene expression in the STR shows the CAR-dependence for transduction of SNc dopaminergic neurons by Ad delivered to the ventral midbrain. (**A**) Representative flattened confocal z-stacks through a 35 µm section of the dorsolateral STR in three wt and hCAR animals. GFP-filled axons projecting from the SNc show the level of neuronal transduction that is augmented by the addition of hCAR transgene. (**B**) Densitometric quantification of individual confocal z-planes shows a significant increase in Ad5 transduction capacity in hCAR mice. (**C**) Drawing of a sagittal section through the mouse brain shows the location of vector delivery in the SNc and the location of transgene expression analysis in the STR, approximately 4.25 mm distal from the site of injection. Scale bar in **A** = 150 µm, Green  =  GFP. Box plot in **B** depicts sample minimum, 1^st^ quartile, median, 3^rd^ quartile and sample maximum. Statistical significance assigned using two-tailed Student's *t*-test (* indicates P<0.0001). LV  =  left ventricle, MFB  =  medial forebrain bundle.

Direct quantitation of the number of neurons in the SNc infected by Ad5 proved impossible due to the extent of GFP expression in surrounding non-neuronal cells, particularly in the core of the injection location where expression was most robust (**Figure 1**). To quantify the effect of hCAR expression on the transduction of the dopamine cells, we instead examined the anterograde labeling of dopaminergic axons in the dorsolateral striatum, the primary target of the SNc neurons [28], [29], [30]. Since GFP is a soluble protein and diffuses freely within the intracellular space, these distal axons are filled when the infected neuronal soma expresses the transgene. We used confocal imaging of dorsolateral striatal sections stained for GFP and TH, collected image stacks, and measured the density of GFP-positive fibers using a threshold approach as described in the Methods. We found that in the hCAR animals, there was an approximately 100-fold increase in the number of anterogradely labeled fibers in the striatum (Percentage of total area occupied, mean ± SEM of wt  = 0.05±0.002; hCAR = 5.1±0.3), consistent with increased transduction of TH neurons in the SNc (**Figure 4**).

### CAR Expression in wild type and hCAR transgenic brain

**Figure 5:**
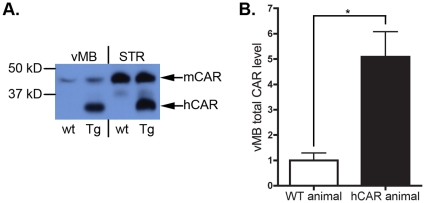
CAR expression in wild type and hCAR transgenic brain. Regional analysis of CAR protein shows variability in expression levels, which are elevated in hCAR transgenic animals. (**A**) A representative immunoblot of CAR protein levels in the ventral midbrain (vMB) and striatum (STR) shows large differences in expression. The truncated transgenic hCAR protein is seen running with a lower apparent molecular mass. Both mCAR and hCAR are visualized with the pan-CAR antibody H300. Transgenic hCAR is expressed in both the vMB and STR at a similar level to that of STR mCAR. (**B**) Quantitation of total CAR in the ventral midbrain by combining endogenous mCAR and transgenic hCAR shows five-fold more CAR protein available in this region in hCAR animals. Total CAR protein level of wt animals is considered baseline. Statistical significance assigned using two-tailed Student's *t*-test (* indicates P<0.02).

CAR expression has not been closely examined in the brains of either adult wild type mice or hCAR transgenic animals [26], [27], [31]. We therefore performed western blot analysis using an antibody that recognizes both human and mouse CAR (Santa Cruz, sc-15405), and observed substantial regional variation in the abundance of endogenous mCAR protein in adult wild type mice. There was strong expression of mCAR in the striatum in wild type mice, while expression in the ventral midbrain (vMB, containing the SN) was much lower, about one fifth of that in the striatum (**Figure 5A**). In the hCAR transgenic mouse, a truncated version of the hCAR protein is encoded by the transgene, resulting in a protein with lower molecular mass than that of endogenous mCAR. This difference in size allowed us to analyze separately the hCAR and the endogenous mCAR present in the transgenic animals. We found that in these animals there was abundant expression of hCAR throughout the brain, with high abundance in both striatum and vMB (**Figure 5A, B**), and that the presence of this transgenic protein did not appreciably alter the expression of the endogenous mCAR. Thus, in the hCAR transgenic animals the overall abundance of CAR in the vMB (hCAR together with mCAR) was five fold higher than in the wild type animals.

### Histological analysis of hCAR mice shows transgene expression localizes to post-synaptic sites in SN and STR

**Figure 6:**
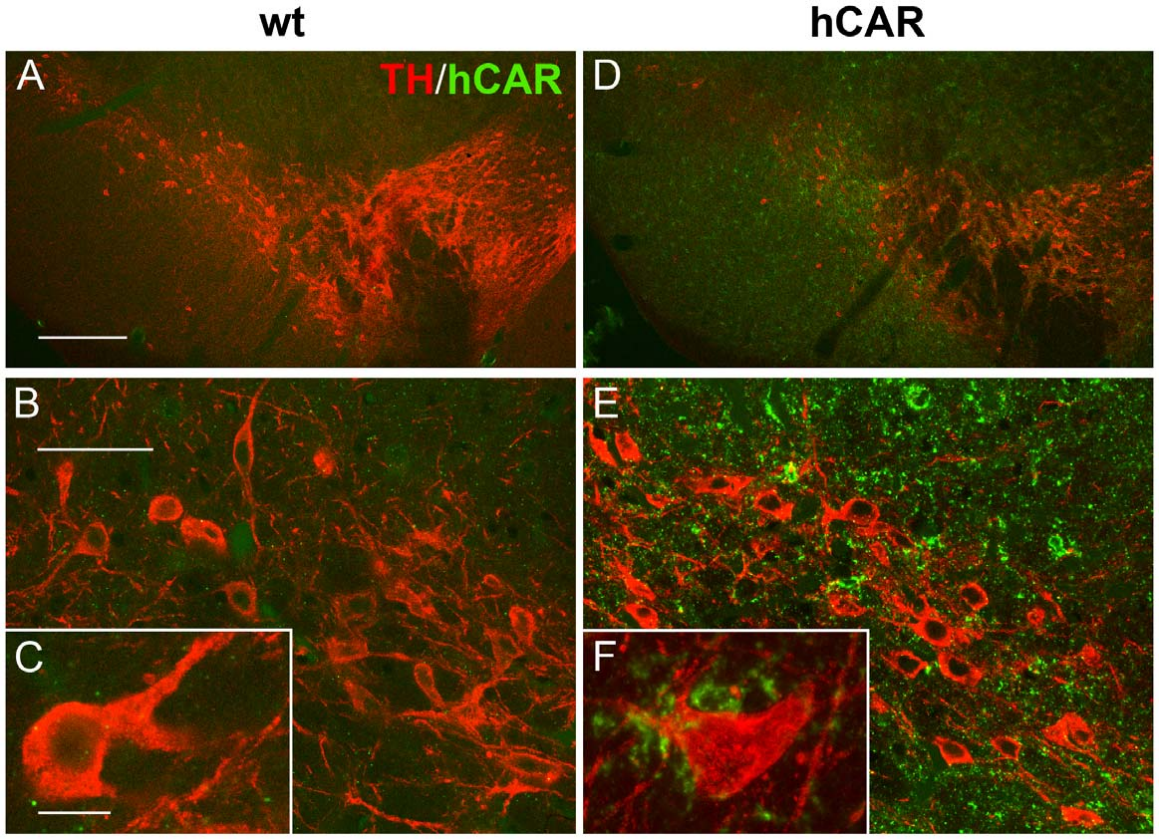
Histological analysis of hCAR localization shows expression in close apposition to SN neurons. Although we were unable to visualize mCAR by immunohistochemistry (data not shown), confocal analysis of hCAR in the substantia nigra of transgenic animals (right panels) showed robust expression throughout the ventral midbrain (**D**). hCAR was readily seen within the SNc (**D, E**), and in close apposition to DA-positive SN neurons (**F**). (**A**–**C**) Left panels show lack of cross-reactivity of antibody RmcB for mCAR. Scale bar **A**, **D**: 250 µm, **B**, **E**: 50 µm, **C**, **F**: 10 µm. Red  =  TH-positive neurons, Green  =  hCAR.

**Figure 7:**
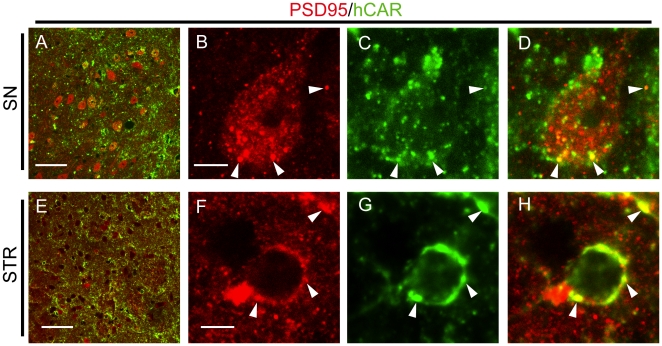
Transgenic hCAR localizes to post-synaptic structures. Assessing the expression pattern of exogenous hCAR in the SN and STR of transgenic animals, we found co-localization with the post-synaptic density marker PSD95 (**A–H,** closed arrowheads indicate areas of co-localization). SN =  substantia nigra, STR  =  striatum. Bars in **A** and **E** = 50 µm, bars in **B** and **F** = 5 µm.

Although mCAR and hCAR were readily detectable via western blot in the transgenic hCAR animals, histological imaging of endogenous mCAR proved problematic with the CAR antibodies and amplification techniques available. We were able to perform immunohistochemistry for hCAR in the vMB to identify at a cellular level the localization of hCAR in this region, allowing us to understand the role this exogenous protein plays in expanding Ad transduction, and better understand the utility of tropism-modified ADs. Expression of hCAR was seen throughout the vMB including within the SNc. Interestingly, most of the staining was punctate, and did not associate with cellular elements. Much of the punctate hCAR staining was closely apposed to TH-positive neurons (**Figure 6A–B, D–E**), but hCAR was not detected within the TH-positive neurons themselves (**Figure 6C, F**). We studied the cellular localization of the hCAR protein in the transgenic animals in more detail using dual-label immunohistochemistry. hCAR was not seen to localize with the astrocyte marker ALDHL1 in the SN (**Figure S1 A, B**), nor with the neuronal marker MAP2 in the cortex, SN or STR (**Figure S1 C–H**). Both of these experiments suggested that the bulk of the hCAR protein was located not in cellular perikarya, but rather within the neuropil. Using an antibody for the protein PSD-95, which is found at post-synaptic sites and within dendritic spines, we observed extensive colocalization of PSD-95 with hCAR in both the SN and STR (**Figure 7A–H**). In contrast, co-staining with the pre-synaptic marker synaptophysin revealed that hCAR staining was often closely opposed to, but not overlapping with, synaptophysin-positive puncta (**Figure S1 I–L**).

## Discussion

The goal of this study was to assess the utility of adenoviral vector systems as platforms for CNS gene therapy in Parkinson disease, and to investigate the factors that modulate the ability of Ad vectors to deliver therapeutic cargos to dopamine neurons in the SNc. We have found that native, Ad5 vectors are inefficient in delivery of genes to SNc dopamine neurons in wild type mice. The efficiency of delivery is greatly enhanced in animals with transgenic expression of hCAR, leading to a nearly 100-fold increase in the density of labeled nigrostriatal axons. Western blot analysis confirmed that the hCAR animals have a much higher level of CAR in the region of the SNc. Dual-label studies suggest that the transgenic protein in these hCAR animals is concentrated at postsynaptic sites on neurons. Together these data indicate that tropism and CAR dependence are critically important in controlling the ability of Ad vectors to deliver genes to dopamine neurons, and that synaptic sites may be important for entry of the virus.

The observation that CAR is central to regulating tropism in the CNS is consistent with prior studies in non-CNS tissues. The capability of Ad5 to infect specific cell types is correlated to cellular CAR status, and of direct relevance, CAR-independent Ad vectors overcome this transductional limitation [22], [32], [33]. As CAR functions in cellular adhesion, cancers are frequently deficient in this protein and therefore refractory to Ad5 transduction. Development of tropism modified, CAR-independent Ad vectors has therefore played an important role in allowing the pre-clinical development of novel tumor-targeted Ad vectors [34], [35], [36], [37]. The first clinical trial evaluating a tropism-modified Ad vector containing a genetically modified fiber protein, allowing Ad transduction of cancer cells deficient in CAR (as wt SNc neurons have herein been shown) has recently been completed and suggests that this paradigm is safe for use in patients [38], and the development of other such trials is continuing [39]. The ongoing development of fiber-modified, CAR-independent forms of Ad5 has greatly expanded the tropism and therefore utility of this vector system, but these novel Ad vectors have yet to be assessed in the context of targeting in the CNS for neurodegenerative diseases, including Parkinson disease.

We used immunohistochemical methods to localize hCAR in the SN of transgenic animals, and found that the majority of the hCAR protein localizes to post-synaptic structures in the neuropil and along the margins of neurons while relatively little is found within neuronal or glial perikarya. This finding is interesting since it is known that rabies virus infects neurons through interactions with the synapse [40]. In wild type mice, however, our western blot studies suggest that the overall abundance of mCAR in the SN is quite low, and we were unable to detect the endogenous mCAR protein by immunohistochemistry in the SN. Together these observations suggest that the relative paucity of endogenous CAR accounts for the inability of Ad5 to infect dopaminergic neurons in wild type mice, and that in the hCAR transgenic animal the entry of the virus is facilitated by synaptically-localized CAR protein. In the context of the design of gene therapy strategies, induction of CAR expression is probably not feasible, but development of Ad viruses with modified tropism is a promising approach, and a search for Ad viruses which might interact with other proteins known to be at synaptic sites might prove fruitful.

In these studies, we employed the promiscuous cytomegalovirus (CMV) promoter in order to visualize gene expression in all cell types transduced by the Ad5 vector when delivered directly to this region. Ad vectors do allow for the use of tissue-selective promoters to target gene expression, and it is likely that use of a neuron-restricted or DA-specific promoter element (synapsin-1 or DAT) would have reduced the off-target gene expression in glial and other non-neuronal cells. On the other hand, transcriptional restriction would probably not increase the number of TH neurons labeled in the wild type animals beyond the limited infectivity we observed. Thus, efficient and selective transduction of dopamine neurons will likely require both transductional and transcriptional (double) targeting.

There is an important need for enhanced vector systems in PD gene therapy. To date, clinical trials in PD have used AAV or lentiviral vectors. Overall, these approaches have proven relatively safe and are well tolerated, although evidence for clinical effectiveness in controlled trials is still lacking [1], [4], [5], [10]. One of the key issues contributing to the lack of efficacy in the trials conducted so far is thought to be the relatively low doses of vector used in many of the initial trials. Studies with higher doses are planned, but there is concern that this may unmask an increasing range of adverse effects related to delivery to non-targeted regions. As an example, recent studies in animal models have suggested that direct trophic factor delivery to the SN via high doses of AAV vectors leads to adverse effects on appetite and feeding, perhaps through aberrant transduction of non-target neurons in adjacent hypothalamic nuclei [41], [42], [43] (abstract #186). These findings reinforce the importance of precise targeting when genetically delivering highly active therapeutic proteins [44], [45], [46], [47].

Our studies highlight the importance of CAR protein in regulating the transduction of CNS neurons by Ad5 vectors, and suggest that modification of vector tropism to target CAR-deficient cells can achieve more efficient delivery of therapies to these neurons. Here we achieved increased gene delivery by transgenic overexpression of CAR, a strategy that is suitable only for establishing proof-of-concept in animal models. The most promising alternative is the use of modified forms of Ad, which do not depend on CAR for their cellular tropism. As noted above, modified vectors of this kind have proven therapeutically useful in treating a variety of non-CNS diseases in many model systems. It will be important to investigate such tropism-modified Ads to determine whether they can provide enhanced gene delivery to SNc dopamine neurons in wild-type animals expressing natively low levels of CAR, perhaps through other proteins present at post-synaptic sites. As transductional and transcriptional targeting modifications are incorporated, Ad promises to develop as a flexible platform for the delivery of novel therapeutics, allowing targeted and controlled delivery, while minimizing side effects.

## Materials and Methods

### Ethics Statement

All animal procedures were approved by the Institutional Animal Care and Use Committee of the University of Alabama at Birmingham (UAB Animal Welfare Assurance Number: A3255-01; Study approval number: 100208706) and conducted in accordance with their and National Institutes of Health guidelines.

### Subjects and Surgical Procedure

Mice were housed under 12 hour light/12 hour dark cycle and were provided standard mouse diet. Sixteen wild type C57B/6 mice and eight hCAR transgenic C57B/6 mice (a generous gift from Dr. S Pettersson, Karolinska Institute, Stockholm, Sweden) received vector delivered to the unilateral SN. For experiments outlined in Figure 3, these animals were aged between 16–24 weeks. Transgenic mice express the human CAR gene lacking the cytoplasmic tail, under control of the human ubiquitin-C promoter, allowing hCAR to be expressed in a variety of tissues including the liver, kidney, heart, lungs, brain, and muscle [27]. Under isoflurane anesthesia, the mice were injected stereotaxically with Ad5 into the right SN; coordinates were anterior-posterior, −3.2 mm from bregma, medio-lateral, −1.2 from midline and dorso-ventral, −4.6 from the dura. Mice received 2.5×10^8^ vp/animal in a volume of 2 µl at a flow rate of 0.25 µl/minute over eight minutes, followed by two minutes for diffusion before the syringe was slowly removed.

### Adenovirus vector

The Ad5 vector used in this study was replication deficient (E1 deleted), containing two bicistronic reporter gene cassettes driving GFP and firefly luciferase off separate cytomegalovirus promoters, as previously described (A kind gift from Dr. Wu, University of Alabama at Birmingham) [22].

### Immunohistochemistry

Two weeks post-injection, animals were deeply anesthetized and transcardially perfused with 4% paraformaldehyde in phosphate buffered saline (PBS). Brains were removed and post-fixed in the same fixative for two hours at room temperature before cryopreservation by impregnation with 30% sucrose in PBS, approximately two days at 4°C. Tissue was flash frozen in isopentane on dry ice and 40 µm sections were cut using a sliding microtome and collected as free-floating tissue in PBS:Glycerol, 1∶1. For GFP and TH staining, floating sections were blocked with 10% normal goat serum for 60 minutes followed by incubation with mouse anti-GFP 1∶20,000 (MAB3580, Millipore, Billerica, MA) and rabbit anti-TH 1∶2000 (P40101-0, PelFreez Biologicals, Rogers, AR) for 48 hours at 4°C. Tissue was then washed in PBS, followed by incubation in a 1∶500 dilution of alexa-488 conjugated goat anti-mouse (Molecular Probes, Carlsbad, CA) or CY3-conjugated goat anti-rabbit (Jackson Immunoresearch, West Grove, PA) secondary antibodies. Slides were then coverslipped with Vectashield mounting medium (Vector Labs, Burlingame, CA). For DAB chromogenic staining technique, tissue was incubated in 3% H_2_0_2_ in methanol for 10 minutes before application of primary antibody. Biotinylated secondary goat anti-mouse was used at 1∶2000 dilution, followed by amplification with the ABC system (PK-7100, Vector Labs) and chromogenic precipitation with DAB (SK-4100, Vector Labs).

To visualize hCAR protein, we utilized tyramide signal amplification (TSA, Perkin Elmer, Waltham, MA), on the anti-CAR monoclonal antibody RmcB (05-644, Millipore, Billerica, MA) following manufacturer's recommended instructions. Briefly, peroxidase is quenched with 3% H_2_O_2_ in methanol, tissue is washed, and blocked with supplied buffer, 30 minutes. TH, ALDHL1 (1∶250, Abcam, Cambridge, MA), MAP2 (1∶500, Abcam), Synapsin (1∶500, Dako, Carpinteria, CA), PSD95 (1∶100, Santa Cruz Biotechnology, Santa Cruz, CA), and RmcB (1∶600 dilution) primary antibodies are incubated overnight at 4°C. Tissue is then washed and secondary antibody is applied (for RmcB, HRP-conjugated goat anti-mouse, 1∶500) for 60 minutes at room temperature. Tissue is washed, and fluorescein amplification reagent is incubated 8 minutes. Tissue is washed and coverslipped with Vectashield aqueous mounting medium.

### Imaging

Confocal images were captured using a Leica TCS-SP5 laser scanning confocal microscope. The images were processed using the Leica software and exported as Tiff files and processed using Adobe Photoshop CS3. For quantitation of percent area occupied, the threshold approach was utilized. All tissue analyzed was stained simultaneously and Z-stacks (2 µm step size) of GFP-stained sections through the dorsolateral STR were obtained with identical confocal settings. These files were then loaded into the ImageJ software package, where stacks were converted into 8-bit grayscale images. A threshold level of 83–255 was set for all images, and percent area was measured. Statistics were calculated using two tailed Student's *t*-test.

### Immunoblotting

Tissue was rapidly extracted from freshly sacrificed animals, dissected to subregions, immediately flash frozen at −80°C in isopentane on dry ice and stored at −80°C. To prepare for immunoblotting, cells were dissociated in homogenization buffer (320 mM sucrose, 10 mM Tris-HCL, 5 mM EDTA, complete protease inhibitor cocktail/PhosSTOP phosphatase inhibitor cocktail, Roche Applied Science) using a tissue homogenizer briefly at medium setting. A cell-intact pellet was obtained by centrifugation at 15000 rpm at 4°C for 16 minutes. Pellet was resuspended in TE buffer (10 mM Tris-HCL, 5 mM EDTA, pH 7.4) and sonicated on ice for 10 seconds. Protein concentrations were then determined by the bicinchoninic acid assay (BCA) and samples were diluted to a final concentration of 1 mg/ml in TE buffer. Boiled samples were mixed with 2×reducing stop buffer (0.25 M Tris–HCl, pH 6.8, 5 mM EDTA, 5 mM EGTA, 25 mM dithiothreitol, 2% SDS, 10% glycerol, and bromophenol blue as the tracking dye), boiled for 10 minutes, and run on 8% SDS-polyacrylamide gels before transfer to PVDF membrane. Blots were blocked in 5% nonfat dry milk in TBST (20 mM Tris–HCl, pH 7.6, 137 mM NaCl, 0.05% Tween 20) for 1 hour at room temperature, then incubated with anti-CAR antibody (H300, Santa Cruz Biotechnology, Inc., Santa Cruz, CA) overnight at 4°C. Membranes were then washed three times with TBST and incubated with HRP-conjugated secondary antibody for 1 hour at room temperature. The membranes were rinsed 6 times for 10 min with TBST, and developed with the enhanced chemiluminescence method. Optical density was quantitated with ImageJ software package and statistics was calculated using two tailed Student's *t*-test.

## Supporting Information

Figure S1 hCAR transgene expression does not localize with astrocytes, neurons, or the pre-synaptic structures. hCAR does not co-localize with the astrocyte marker ALDHL1 in the SN (A, B), nor does it localize to neuronal dendrites or cell bodies (MAP2 staining, C–H). Staining for the pre-synaptic marker synaptophysin showed close association of staining with hCAR (I–L, closed arrowheads indicate adjacent areas). SN  =  substantia nigra, CTX  =  cortex, STR  =  striatum. Bars in A and E  = 100 µm; bars in C and G  = 50 µm; bars in B, D, F, H  = 20 µm; bar in I  = 10 µm; bar in J for J–L  = 2 µm.
(8.32 MB TIF)
