# Supplementary figures and images for: Transduction of Brain Dopamine Neurons by Adenoviral Vectors Is Modulated by CAR Expression: Rationale for Tropism Modified Vectors in PD Gene Therapy
Source: PLoS One. 2010 Sep 17;5(9):e12672. doi: 10.1371/journal.pone.0012672 (PMC2941453; doi:10.1371/journal.pone.0012672)

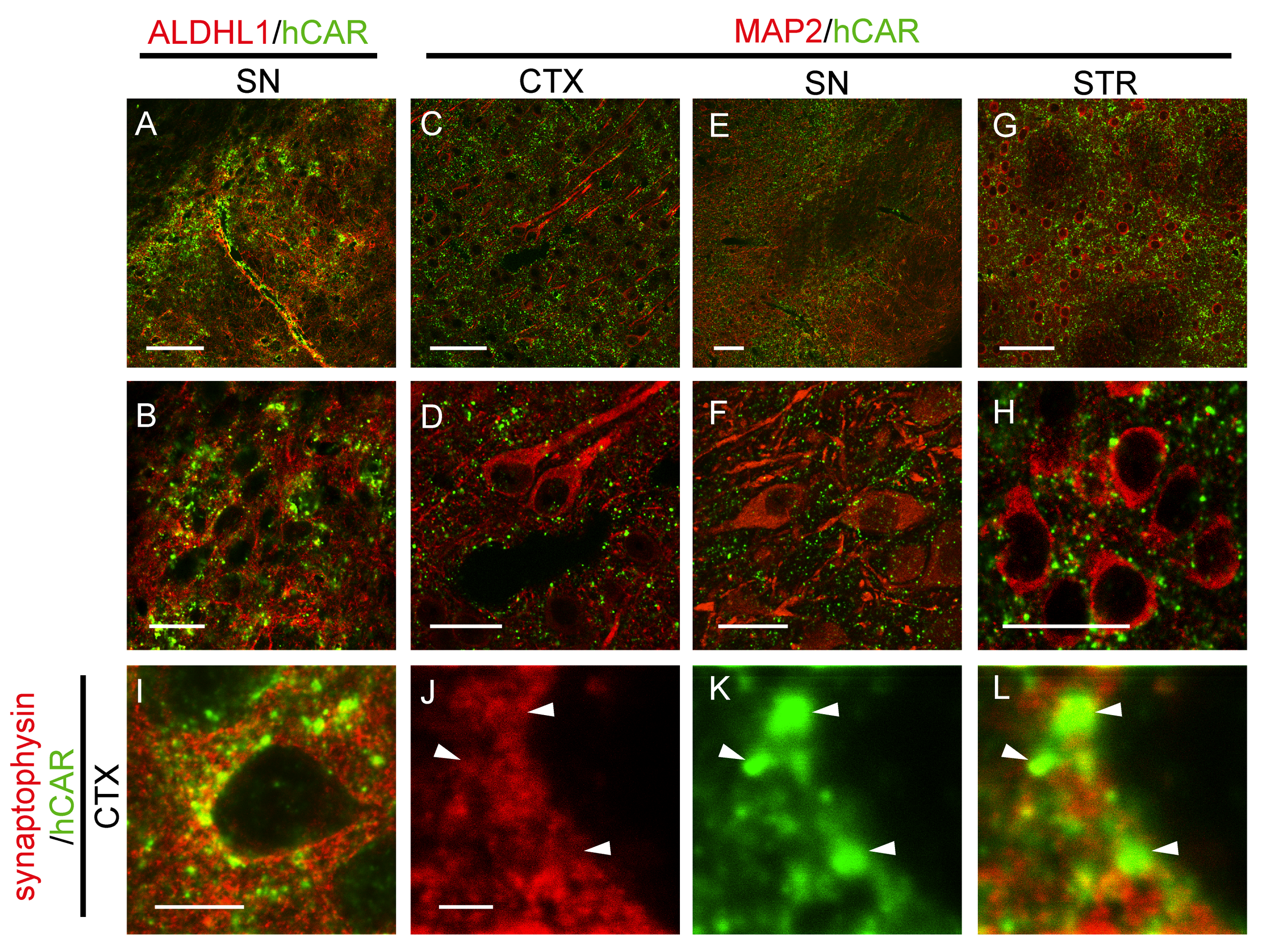

Supplement: Figure S1 — hCAR transgene expression does not localize with astrocytes, neurons, or the pre-synaptic structures. hCAR does not co-localize with the astrocyte marker ALDHL1 in the SN (A, B), nor does it localize to neuronal dendrites or cell bodies (MAP2 staining, C–H). Staining for the pre-synaptic marker synaptophysin showed close association of staining with hCAR (I–L, closed arrowheads indicate adjacent areas). SN = substantia nigra, CTX = cortex, STR = striatum. Bars in A and E = 100 µm; bars in C and G = 50 µm; bars in B, D, F, H = 20 µm; bar in I = 10 µm; bar in J for J–L = 2 µm. (8.32 MB TIF) [file pone.0012672.s001.tif]
